# A Case of Typhoid Fever, with Comments

**Published:** 1889-01

**Authors:** E. J. Lee


### A CASE OF TYPHOID FEVER, WITH COMMENTS.

In reporting this case of typhoid fever, I have nothing very new to offer; the case is reported to illustrate the use of a remedy not frequently called for in this disease, and further to show the wonderful curative powers of our drugs, when properly given, even in the most extreme cases.

March 6th, 1888.—Was called to see E. F. W., aged eighteen; a tall, slim young man with a badly developed cranium, it being almost flat on the sides. He had been complaining of various aches and pains for some days, perhaps for two weeks. For these prodromic symptoms of typhoid fever he had been treated by an allopath. The young man was away from home at school; his mother was immediately summoned; on her arrival, seeing the gravity of the case, she telegraphed to her family physician, Dr. Elliott, of Brooklyn, to recommend a Philadelphia physician. He kindly sent her my name.

When I first saw the patient, he had been in his bed for three days, and, what was much worse, for ten days under the Gatling-gun-like prescribing of this country allopath! Fortunately for the young man, his mother was a staunch believer in Homœopathy; had he remained a few days longer under the scientific treatment of the regular, his end would assuredly have been death. Before I called he had given the principal of the school to understand that there was very little hope of recovery.

However, the allopath very kindly consented to meet me, and he gave me an outline of the case; premising it by the statement that the patient would not respond to his medicines. He was honest at least; he probably meant to say that his medicines would not act as he desired them to act. But, as he had been practicing for a good many years, he ought to have gotten accustomed to this, as their drugs never do act as they are labeled to act. He said he had given the boy, in the last few days, Morphia, Quinia, the Bromides of Sodium and Potassium, and lastly the tincture of Jaborandi had been given, since five A. M. that morning, for the purpose of making the patient sweat. The boy's mother told me he had been growing worse since taking the Jaborandi, had had no sweat nor any urine. At the time of my visit the following was the condition of the patient. No urine for some hours, exact number could not be ascertained; the patient was lying on his back; his face was very pale; it was hot and dry; the tongue thickly coated, white in the centre, edges and tip red, also dry and cracked; patient could scarcely protrude tongue, when asked to do so, it trembled and oscillated; had to be told to put it back again. The teeth and lips were covered with sordes; the mouth was wide open; the patient was in a stupor, totally unconscious of all that was going on around him; could be aroused, but would immediately sink again into this stupor. The urine had been passed unconsciously, before its suppression.

The worst feature of the case was the violent convulsive movements of the limbs; the feet and hands were continually jerking and twitching; the motion of the hands was peculiar. The elbows rested on the bed, the arms and hands waved and jerked from the rested elbows as from a fulcrum, the finger-tips were bunched together and the wrists were flexed on the forearm and slightly rotated. The movements were continuous and violent, unconsciously preformed; *but they ceased whenever the patient seemed to be sleeping.* The eye-lids were half open; the eye-balls were turned up, so that only the whites could be seen, and were immovable; the pupils were dilated and insensible. On lifting the patient, to arrange the bed, it was discovered that his back and neck were so rigid that he could be lifted up by the head without a bend from head to feet.

This condition, together with the previous dosing, did not indicate a very favorable prognosis. The appearance of the patient's face, the *cessation of the convulsive movements on his sleeping*, and the peculiar choraic-like motions, led me to give him Agaricus$^{2c}$, four doses in water. The first dose was given about twelve; at quarter of one P. M., he passed urine, or, as his mother expressed it, he just flooded the bed. This was, of course, an evidence of improvement.

March 7th.—At nine A. M., found the boy conscious; that he had had frequent passages of both stool and urine during the night, both involuntary. The jerkings were less frequent and violent. The stools were liquid, but of natural color and odor; the temperature and pulse were improved. Gave Sac. lac. every hour; milk the only nourishment allowed. The allopath had been giving whisky and milk "to keep up the strength."

March 12th.—Patient improved steadily during last five days, the urine became lighter in color, the bowels moved less frequently and stools were more solid; temperature lower, the pulse firmer and slower. Small boils forming on lip, over sacrum, and on leg.

March 13th.—Found patient had been more feverish, with greater delirium. Tongue was heavy coated and dark in the centre; the lip, in right corner, was much swollen from the boil. Boils on leg and over sacrum were increasing in size and number; action of bowels had changed to a morning diarrhœa of watery stools. The relapse from previous improvement, the morning diarrhœa, the boils, and the drowsiness led me to give one dose of Sulphur$^{cm}$.

March 14th.—Patient no better; gave another dose of Sulphur$^{cm}$.

March 15th.—Mother says patient has been worse since last visit, has been delirious most of the time, but the delirium is of mild type. Lip is less swollen, tongue gives signs of clearing, and is more moist; had three pappy stools; urine clearer and more profuse.

March 16th.—Found patient improved; temperature lower, pulse again firm, less delirium; tongue clearer and more moist; since last visit has had one or two spells of vomiting of sour milk with a little blood in it. The boils seem to be drying up. Continued the Sac. lac. and milk. The second dose of Sulphur was an error; it served only to retard improvement. From this date the patient slowly improved in every way, the mind became clearer; the stools and urine more normal.

March 29th.—The patient having been troubled lately by copious night-sweats, and being despondent as to recovery, one dose of Psorinum$^{5c}$ was given, with the effect of stopping the sweats and seemingly establishing convalescence. From March 29th to April 6th, the patient seemed to improve steadily, therefore no medicine was given; the diet was chiefly milk, with an occasional spoonful of Valentine's beef extract, diluted in water. Perhaps from over-feeding or from such cause, a decided relapse was produced. Patient became much worse; had more fever, with great thirst; was very restless, yet complained of being tired all the time; had frequent yellow stools, tongue was dry and red; licked his lips; had red spots on each cheek, was drowsy. Arsenicum$^{5c}$ was given April 6th. This was, as afterward proven, a bad prescription. The patient grew worse for the next two days.

April 9th.—Patient worse; more delirium, greater tendency to comatose sleep; lies with eyes half-open, has delirium on closing the eyes; talks in sleep but can't remember, on waking, anything he has said or dreamt; tongue dry, heavily coated, dark in the centre; has disposition to frequently raise the head from the pillow. For the last two weeks this patient had been troubled by the delusion that there was "another fellow" in bed with him; whenever the bed-pan was used, he would ask that it be given to the "other fellow" too. As the patient was improving when this symptom was first noticed, no change was made in the prescription. It is not given under any of the remedies mentioned so far in this case; had this mental symptom been taken into account when the Arsenicum was selected, probably a better prescription would have been made. This delusion, with the peculiar coating of the tongue, indicated Baptisia, which, as it was at least not contra-indicated by any of the other symptoms, was given. A dose of Baptisia$^{5c}$ was given, one dose on the 9th, and a second on the 10th. After events proved this also to be a bad prescription. Stramonium would probably have been the proper remady; but it is much easier to see our errors after they are made than it is to prevent making them.

April 11th.—Patient had a bad night, high fever, violent delirium, temperature again high; deep coma, patient in almost profound stupor, urine and stool passed involuntarily and unconsciously. As patient had had a copious warm sweat during the early morning hours, it was thought best to wait until evening to see if this sweat would relieve. Called in the evening and found the patient still worse; no urine since early morning; stupor deeper. Decided now that another remedy must be selected. A careful examination of the patient revealed these symptoms: Stupor with delirious muttering; constant raising of the head from the pillow or boring into the pillow, so that he would finally push his head against the head-board of the bed; he would raise his hands to his face, ears, etc., or wave them in the air or scratch on the wall; there was violent twitching of the muscles; rigidity of the back and limbs; the masseter muscles were rigid, clenching the jaws; pupils were dilated, the eyes injected and staring; frowning; moving of lips back and forth; coldness of face, nose, ears, chin, hands, and feet; involuntarily, loose, but small stools; no urine; hot sweat on the body; tongue, when last seen, was red on edges and tip, dry and heavily coated in the centre. The delirium was constant; the patient entirely unconscious, yet he wanted to get up and dress or to walk about the room, etc., fever and delirium worse from four P. M. to midnight. These very grave symptoms, occurring in the sixth week, made a fatal termination very probable. Realizing the gravity of the case, I retired to another room with a repertory and a copy of Hering's *Condensed*. After some study, I concluded that the sole hope of that boy lay in Stramonium, which was accordingly given in the 200th potency, a dose every two hours, six doses in all. The first dose was given about seven P. M.; the first effect of this remedy was to warm up the cold extremities; the second was the passage of a little urine, and the third was the opening of his eyes at six A. M., and asking for a drink. Sac. lac. was now given, with milk as desired.

April 12th.—As the urine was very scanty, another dose of Stramonium, this time the CM, was given; the patient recovered without another dose of anything save Sac. lac. He was five days regaining consciousness; but, from the first dose of Stramonium, given that night, when death seemed so near, until leaving his bed, April 26th, to take the cars for the sea-shore, his improvement was steady and continuous. No stimulants were used; the diet was chiefly of milk, with occasional doses of beef extract, yet this boy was able to travel some forty miles the first day out of his bed; the second day he walked into the dining-room at his hotel for his breakfast, and so on. But I was severely criticised by the allopathic doctor and by his friends at the school, for not using stimulants to give the boy strength.

Thus it will be seen that this case began and ended with suppression of the urine and consequent convulsions. The Agaricus was given for the peculiarities of the patient, and also as the convulsive motions ceased on his going to sleep; Hellebore has this also. It will be noticed that no antidotal remedy was given for the previous dosing; none was given, simply because none was indicated. Opium also covered a great many symptoms of the case; but Agaricus not only covered the symptoms of the disease but also fitted the peculiarities of the patient's constitution. The use of Psorinum, which perhaps stopped too quickly the sweats, may have been an error, for, had these sweats continued, the subsequent relapse might never have occurred.

The use of both Arsenicum and Baptisia, later on, were certainly improper, and came near causing a fatal termination. The action of Stramonium was most prompt and thorough, even in face of such a grave case; for the end of that boy was certainly not far off when those few dried pellets of Stramonium were given him.

E. J. LEE.

